# Palmitoleic Acid Inhibits RANKL-Induced Osteoclastogenesis and Bone Resorption by Suppressing NF-κB and MAPK Signalling Pathways

**DOI:** 10.3390/nu9050441

**Published:** 2017-04-28

**Authors:** Bernadette van Heerden, Abe Kasonga, Marlena C. Kruger, Magdalena Coetzee

**Affiliations:** 1 Department of Physiology, University of Pretoria, Pretoria 0001, South Africa; bernadettevanheerden@ymail.com (B.v.H.); abe.kasonga@up.ac.za (A.K.); 2 School of Food and Nutrition, Massey Institute of Food Science and Technology, Massey University, Palmerston North 4442, New Zealand; m.c.kruger@massey.ac.nz; 3 Institute for Food, Nutrition and Well-being, University of Pretoria, Pretoria 0001, South Africa

**Keywords:** palmitoleic acid, osteoclasts, bone resorption, fatty acid

## Abstract

Osteoclasts are large, multinucleated cells that are responsible for the breakdown or resorption of bone during bone remodelling. Studies have shown that certain fatty acids (FAs) can increase bone formation, reduce bone loss, and influence total bone mass. Palmitoleic acid (PLA) is a 16-carbon, monounsaturated FA that has shown anti-inflammatory properties similar to other FAs. The effects of PLA in bone remain unexplored. Here we investigated the effects of PLA on receptor activator of nuclear factor kappa B (NF-κB) ligand (RANKL)-induced osteoclast formation and bone resorption in RAW264.7 murine macrophages. PLA decreased the number of large, multinucleated tartrate resistant acid phosphatase (TRAP) positive osteoclasts and furthermore, suppressed the osteolytic capability of these osteoclasts. This was accompanied by a decrease in expression of resorption markers (*Trap*, matrix metalloproteinase 9 (*Mmp9*), cathepsin K (*Ctsk*)). PLA further decreased the expression of genes involved in the formation and function of osteoclasts. Additionally, PLA inhibited NF-κB activity and the activation of mitogen activated protein kinases (MAPK), c-Jun N-terminal kinase (JNK) and extracellular signal–regulated kinase (ERK). Moreover, PLA induced apoptosis in mature osteoclasts. This study reveals that PLA inhibits RANKL-induced osteoclast formation in RAW264.7 murine macrophages through suppression of NF-κB and MAPK signalling pathways. This may indicate that PLA has potential as a therapeutic for bone diseases characterized by excessive osteoclast formation.

## 1. Introduction

Bone resorption and formation are tightly coupled in healthy individuals in a process known as bone remodelling [1]. However, when there is an imbalance between formation and resorption, many bone pathologies may arise such as osteoporosis, osteopetrosis, and rheumatoid arthritis [2]. There are three main bone cell types involved in bone remodelling namely; osteocytes; osteoblasts, and osteoclasts. Osteocytes remain embedded within the mineralized matrix of bone and are thought to translate mechanical loading into biochemical signals that affect bone remodelling [3]. Osteoblasts are responsible for the synthesis and mineralization of bone as well as modulating the differentiation of osteoclasts [4]. Osteoclasts are large, multinucleated cells that are responsible for the resorption of bone. They are formed by the fusion of mononuclear hematopoietic cells of the monocyte-macrophage lineage in the presence of differentiation factors, macrophage colony-stimulating factor (M-CSF) and receptor activator of nuclear factor kappa B (NF-κB) ligand (RANKL), both produced by osteoblasts [5,6].

Osteoclasts resorb bone by developing a microenvironment in which the mineral part of the bone is dissolved and the organic part is degraded [7]. The osteoclast forms a sealing zone and creates a tight seal with the matrix of the bone. This sealing zone encircles the ruffled border responsible for increasing the surface area for resorption [7]. Binding of RANKL to the receptor activator of nuclear factor kappa B (RANK) on osteoclast precursors will trigger the NF-κB pathway and the mitogen activated kinase (MAPK) pathways, c-Jun N-terminal kinase (JNK), extracellular signal–regulated kinase (ERK), and p-38. Activation of these pathways will promote the expression of nuclear factor activator T-cells, cytoplasmic 1 (NFATc1) through Fos proto-oncogene (cFos). NFATc1 is known as the master regulator of osteoclast formation and function. NFATc1 will increase expression of dendritic cell-specific transmembrane protein (DC-STAMP), which plays an important role in osteoclast formation through cell-to-cell fusion; the release of collagenolytic enzymes (matrix metalloproteinase 9 (MMP-9), cathepsin K (CTSK), and tartrate resistant acid phosphatase (TRAP)); and the acidification of the resorption lacuna through carbonic anhydrase 2 (CAR2). Long-chain polyunsaturated fatty acids (LCPUFAs) such as alpha-linolenic acid (ALA), docosahexaenoic acid (DHA), and eicosapentaenoic acid (EPA) have been studied for their protective effects on bone health [8]. Studies have shown that these fatty acids (FAs) can increase bone formation, reduce bone loss, and influence total bone mass [9]. EPA and DHA have further been shown to decrease osteoclast formation and activity [8,10,11,12], possibly explaining their bone protective effects. Similar anti-osteoclastogenic effects have been reported in vitro with the use of oleic acid (OA), an anti-inflammatory monounsaturated fatty acid (MUFA) [13].

Palmitoleic acid (PLA) is an omega-7, 16-carbon, MUFA obtained from dietary sources, such as macadamia oil, and can be synthesized endogenously by adipocytes [14,15]. PLA is formed by the dehydrogenation of palmitic acid—a saturated 16-carbon fatty acid. PLA has been shown to have anti-inflammatory properties similar to other fatty acids such as DHA, EPA, and OA [14]. Several studies on PLA have revealed a wide array of effects on health [15,16,17,18]. Results from these studies have shown that PLA downregulates and inhibits lipogenesis, therefore controlling fat production in bovine adipocytes; increases low-density lipoprotein-cholesterol and lowers high-density lipoprotein-cholesterol, but also reduces total cholesterol levels in hypercholesterolemic men; increases basal glucose uptake in skeletal muscles of rats by increasing the number of glucose transporter (GLUT) 1 and GLUT4 transporters in the plasma membrane, hence controlling insulin resistance; and reduces the accumulation of hepatic lipids in mice with Type 2 diabetes [15,16,17,18]. Further studies have found that PLA increases insulin sensitivity and is a strong predictor of insulin sensitivity in humans [19]. However, the effects of PLA on insulin sensitivity have proven controversial, as other studies have revealed contradictory results [20,21]. These conflicting studies underlie the need for further research into the effects of PLA on health. Despite its anti-inflammatory properties and other health benefits, the effects of PLA on bone are largely unexplained. In this study, we investigated the effects of PLA on RANKL-induced osteoclast formation in RAW264.7 murine macrophages. To the best of our knowledge, no previous studies have been conducted on the effects of PLA in osteoclasts.

## 2. Materials and Methods 

### 2.1. Reagents and Materials

Dulbecco’s Modified Eagle’s medium (DMEM) was purchased from GIBCO (Invitrogen Corp, Carlsbad, CA, USA) and heat inactivated fetal bovine serum (FBS) was bought from Amersham (Little Chalfont, UK). Palmitoleic acid (PLA) was acquired from Cayman Chemical Company (Ann Arbor, MI, USA). RANKL was obtained from Research and Diagnostic Systems (R&D Systems, Minneapolis, MN, USA) while TRI Reagent, Trypan blue, and all other chemicals of research grade were obtained from Sigma-Aldrich Inc. (St. Louis, MO, USA). The Alamar blue reagent, 4–12% NuPAGE Novex Bis-Tris polyacrylamide gels, cell extraction buffer, iBlot Gel Transfer Device and the iBlot Western Detection Chromogenic Kit were all supplied by Life Technologies (Carlsbad, CA, USA). M-MuLV reverse transcriptase was purchased from New England Biolabs (Ipswich, MA, USA). Roche FastStart Essential DNA Green Master was acquired from Roche (Basel, Switzerland). The bicinchoninic acid (BCA) protein assay kit was purchased from Thermo Scientific (Rockford, IL, USA) and the rabbit polyclonal antibodies against glyceraldehyde 3-phosphate dehydrogenase (GAPDH), JNK, pJNK, ERK, and pERK were supplied by Abcam (Cambridge, MA, USA).

### 2.2. Fatty Acid Preparation

PLA was prepared at a stock concentration of 100 mM in ethanol, aliquoted, and stored at −80 °C in the dark until required. The stock solution was then freshly diluted to the required working concentrations of 20, 40, 60, 80, and 100 µM in complete culture medium [8]. The highest concentration of ethanol did not exceed 0.1% during the experiments, and this was used for the vehicle control.

### 2.3. Cell Culture

RAW264.7 murine macrophages (#TIB-71) were purchased from American Type Culture Collection (ATCC, Rockville, MD, USA) and were cultured in a flask with DMEM supplemented with 10% FBS and 1% antibiotic solution containing fungizone (0.25 μg/mL), streptomycin (100 μg/mL), and penicillin (100 μg/mL). The cells were incubated at 37 °C and humidified at 5% CO_2_.

### 2.4. Alamar Blue Assay for Cell Viability

RAW264.7 murine macrophages were plated in a sterile 96-well plate at a density of 5 × 10^3^ cells per well. After 24 h for attachment, the cells were exposed to PLA (20–100 µM) or vehicle (0.1% ethanol) for a further 48 h. At the end of the culture period, cells were assessed for cell viability using an Alamar Blue assay as per the manufacturer’s instructions (Life Technologies). Absorbance values were measured using an Epoch Micro-plate spectrophotometer (BioTek, Winooski, VT, USA) at a wavelength of 570 nm and using 600 nm as the reference [22]. Results are expressed as percentage of control.

### 2.5. Osteoclast Formation and TRAP Activity Assay

RAW264.7 murine macrophages were seeded into a sterile 96-well plate at a density of 5 × 10^3^ cells per well in complete culture medium with increasing concentrations of PLA (20–100 µM) or vehicle (0.1% ethanol) in combination with 15 ng/mL of RANKL for 5 days. The cell culture media and compounds were replaced on day 3. At the end of the culture period, TRAP activity was measured in conditioned media while the cells were stained for the presence of TRAP. Assessment of TRAP activity was done as previously described [8] and was quantified by optical absorbance at 405 nm using an Epoch Micro-plate Spectrophotometer (BioTek). The results are expressed as percentage of the control. TRAP staining of the cells was performed as previously described [8]. TRAP-positive (pink) multinucleated cells that contained more than three nuclei were regarded as osteoclasts and were counted [22]. Photographs were taken of these cells with a Discovery V20 StereoMicroscope using an AxioCam MRc5 camera (Carl Zeiss, Oberkochen, Germany).

### 2.6. Actin Ring Formation

RAW264.7 murine macrophages were seeded into a sterile 96-well plate at a density of 5 × 10^3^ cells per well in complete culture medium with increasing concentrations of PLA (20–100 µM) or vehicle (0.1% ethanol) in combination with 15 ng/mL of RANKL for 5 days. The cell culture media and compounds were replaced on day 3. At the end of the culture period, the cells were fixed with 3.7% formaldehyde solution in PBS for 10 min and then permeabilized with 0.1% Triton X-100 for 10 min. The cells were then stained with Alexa fluor 568 phalloidin (Life Technologies) for 40 min, followed by 35 µg mL^−1^ Hoechst 33342 solution for 10 min to stain the nuclei. Photomicrographs were then taken using a Zeiss inverted Axiovert CFL40 microscope equipped with a Zeiss Axiovert MRm monochrome camera (Carl Zeiss, Oberkochen, Germany). The following filters were used: Hoechst (Excitation: 352 nm, Emission: 455 nm); Phalloidin (Excitation: 502 nm. Emission: 525 nm).

### 2.7. Bone Resorption Assay

RAW264.7 murine macrophages were seeded into a sterile 24-well osteoassay plate (Corning Inc., Corning, NY, USA) at a density of 1 × 10^4^ cells per well, in the presence of RANKL (30 ng/mL) and varying concentrations of PLA (40–100 µM) or vehicle (0.1% ethanol) for 5 days [23]. At the end of the culture period, a modified von Kossa stain was performed and photomicrographs of the resorbed areas were taken as previously described [23]. Resorption was quantified using ImageJ software [24].

### 2.8. Polymerase Chain Reaction (PCR)

RAW264.7 murine macrophages were seeded into a sterile 24-well plate at a density of 1.5 × 10^4^ cells per well and were exposed to PLA (100 µM) or vehicle (0.1% ethanol) in combination with RANKL (15 ng/mL) for 5 days. TRI reagent was used to extract the total cellular RNA and 1 µg of this RNA was reverse transcribed into cDNA using M-MuLV reverse transcriptase. Roche FastStart Essential DNA Green Master was used for the quantitative real time PCR (qT-PCR). Gene expression was analysed using the 2^−ΔΔCT^ method after results were normalized to *Gapdh* [23]. The primers used in this study were synthesized by Inqaba Biotec (Pretoria, South Africa) and are shown in Table 1.

### 2.9. Western Blotting

RAW264.7 murine macrophages were seeded in a sterile six-well plate at a density of 1 × 10^6^ cells per well and incubated at 37 °C for 24 h. Thereafter, the cells were pre-exposed to PLA (100 µM) or the vehicle (0.1% ethanol) for 4 h. Thereafter, cells were exposed to RANKL (15 ng/mL) in combination with PLA (100 µM) or vehicle (0.1% ethanol) for 30 min. The cells were then lysed in ice-cold radioimmunoprecipitation assay (RIPA) buffer that was already supplemented with protease and phosphatase inhibitors. A bicinchoninic acid (BCA) protein assay kit was used to quantify the purified proteins and equal amounts of protein were resolved on a 12% polyacrylamide gel. Proteins were then electrotransferred onto nitrocellulose membranes with Tris glycine transfer buffer containing 192 mM glycine, 25 mM Tris, and 20% methanol and then probed with specific rabbit antibodies at 4 °C overnight. This was followed by incubation with Immun-Star goat anti-rabbit-horseradish peroxidase conjugate secondary antibody (Bio-Rad, Hercules, CA, USA) and a ChemiDoc MP (Bio-Rad) was then used to obtain digital images of the blots.

### 2.10. NF-κB/SEAP Assay

RAW264.7 macrophages were stably transfected with pNiFty2-SEAP (Invivogen, San Diego, CA, USA), an NF-κB-inducible reporter plasmid containing 5× NF-κB repeated transcription factor binding sites as well as a reporter gene—secreted embryonic alkaline phosphatase (SEAP), with GeneCellin transfection reagent [25]. Transfected RAW264.7 murine macrophages were seeded into a sterile 96-well plate at a density of 5 × 10^3^ cells per well in serum-free selection medium supplemented with Zeocin. The cells were exposed to PLA (20–100 µM) or vehicle (0.1% ethanol) in combination with RANKL (30 ng/mL) and incubated for 48 h at 37 °C and 5% CO_2_. After the culture period, the supernatant was transferred to a separate 96-well plate and heated to 65 °C for 10 min to inhibit endogenous alkaline phosphatase. SEAP assay was conducted as per the manufacturer’s instructions.

### 2.11. Mature Osteoclasts

RAW264.7 murine macrophages were seeded into two sterile 96-well plates at a density of 5 × 10^3^ cells per well for 5 days in the presence of RANKL (15 ng/mL) to allow differentiation. The medium was changed on day 3. On day 5, varying concentrations of PLA (20–100 µM) were added to both plates and incubated for 24 h and 48 h, respectively.

#### 2.11.1. LDH Assay

Necrosis causes permeabilisation of cell plasma membranes, which allows for cellular components such as lactate dehydrogenase (LDH) to leak out of these damaged cells [26]. After the culture period, LDH release was determined as previously mentioned [27]. Readings were taken on a spectrophotometer at an optical absorbance of 490–520 nm.

#### 2.11.2. Hoechst Staining

After the culture period, the cells were washed with PBS and stained with Hoechst in the dark for 5 min. Photomicrographs were taken using a Zeiss inverted Axiovert CFL40 microscope equipped with a Zeiss Axiovert MRm monochrome camera using the following filters: Hoechst (Excitation: 352 nm, Emission: 455 nm) (Carl Zeiss, Oberkochen, Germany).

#### 2.11.3. Statistical Analysis

Three independent experiments were performed in 6-fold, unless otherwise stated, and exposed cells were compared to the vehicle control. Results are displayed as the mean ± the standard deviations. Statistical analysis was done by one-way analysis of variance (ANOVA) followed by a Bonferroni post hoc test using GraphPad Prism software. All *p*-values ≤ 0.05 were considered significant.

## 3. Results

### 3.1. The Effect of PLA on Cell Viability

**Figure 1:**
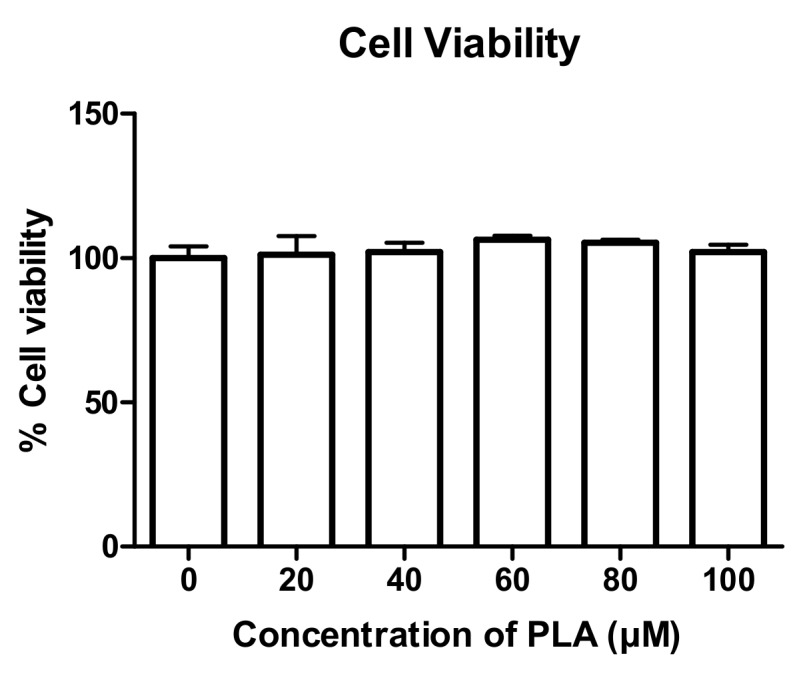
Effect of palmitoleic acid (PLA) on cell viability. Cells were exposed to varying concentrations of PLA (20–100 µM) for 48 h and an Alamar Blue assay was performed to test cell viability. Results are shown as percentage of control and expressed as mean ± standard deviation.

RAW264.7 murine macrophages were exposed to a concentration gradient of 20–100 µM of PLA for 48 h. These concentrations were not found to be cytotoxic to the cells and were used for further testing (Figure 1).

### 3.2. PLA Inhibits Osteoclast Formation

**Figure 2:**
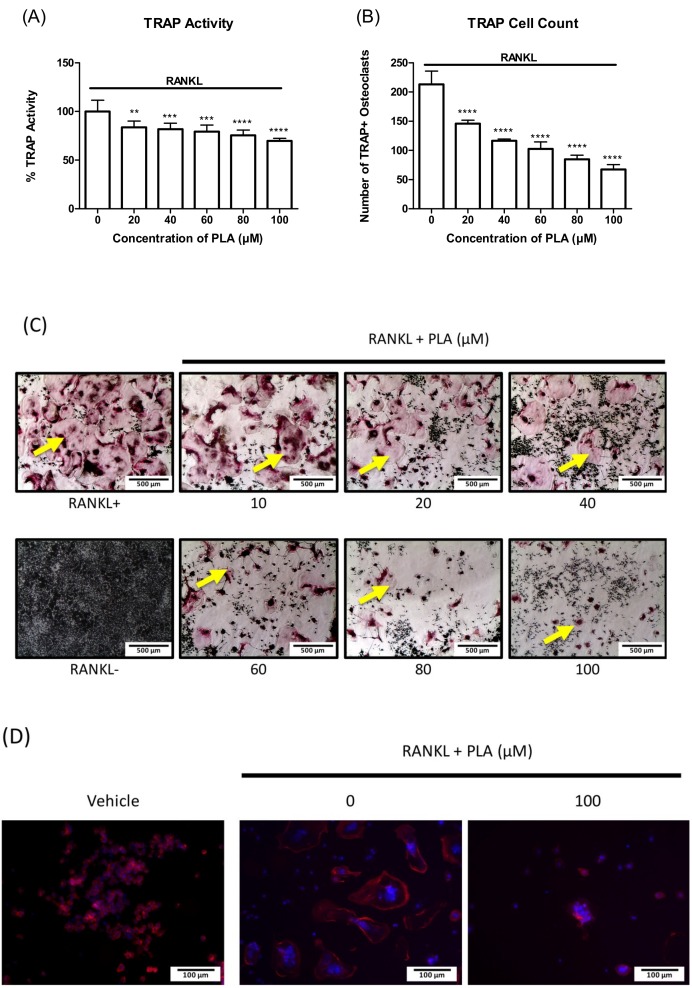
Effect of PLA on tartrate resistant acid phosphatase (TRAP) activity and osteoclastogenesis. RAW264.7 murine macrophages were differentiated into osteoclasts in the presence of receptor activator of NF-κB ligand (RANKL) (15 ng/mL) and PLA (20–100 µM) for five days. (**A**) TRAP activity was determined as described in the methods. Results are shown as percentage of control and expressed as mean ± standard deviation; (**B**) Number of TRAP positive osteoclasts counted at different concentrations; (**C**) Effect of varying concentrations of PLA on osteoclast formation. TRAP positive (pink) osteoclasts are shown by yellow arrows. (Scale bar = 500 µm); (**D**) RAW264.7 murine macrophages were seeded onto glass coverslips with or without of PLA (100 µM) in combination with RANKL (15 ng/mL). Cells were stained for actin (red) and nuclei (blue) using Alexa fluor 568 phalloidin and Hoechst, respectively. (Scale bar = 100 µm). ** *p* < 0.01; *** *p <* 0.001; **** *p* < 0.0001 compared to control.

RAW264.7 murine macrophages were differentiated with RANKL for 5 days in the presence of varying concentrations of PLA. TRAP activity levels (Figure 2A) and number of osteoclasts counted (Figure 2B) were significantly reduced at all concentrations tested. TRAP stain images (Figure 2C) show large, well developed osteoclasts in the RANKL only cells, whereas the size and number of mature osteoclasts reduce with an increase in PLA concentration. The highest reduction was shown at 100 µM, therefore for the PLA concentration of 100 µM only a few multinucleated cells were present. This concentration was used for further experiments.

### 3.3. The Effect of PLA on RAW264.7 Murine Macrophage Morphology

Actin rings were detected by fluorescent microscopy in order to visualize the structure of the actin rings in mature osteoclasts after treatment with PLA. Large multinucleated cells with clear actin rings are seen in wells exposed to RANKL only. PLA reduced the size and number of the osteoclasts with actin rings (Figure 2D). Furthermore, the actin rings that formed are smaller and incomplete after exposure to PLA.

### 3.4. PLA Suppresses Bone Resorption

**Figure 3:**
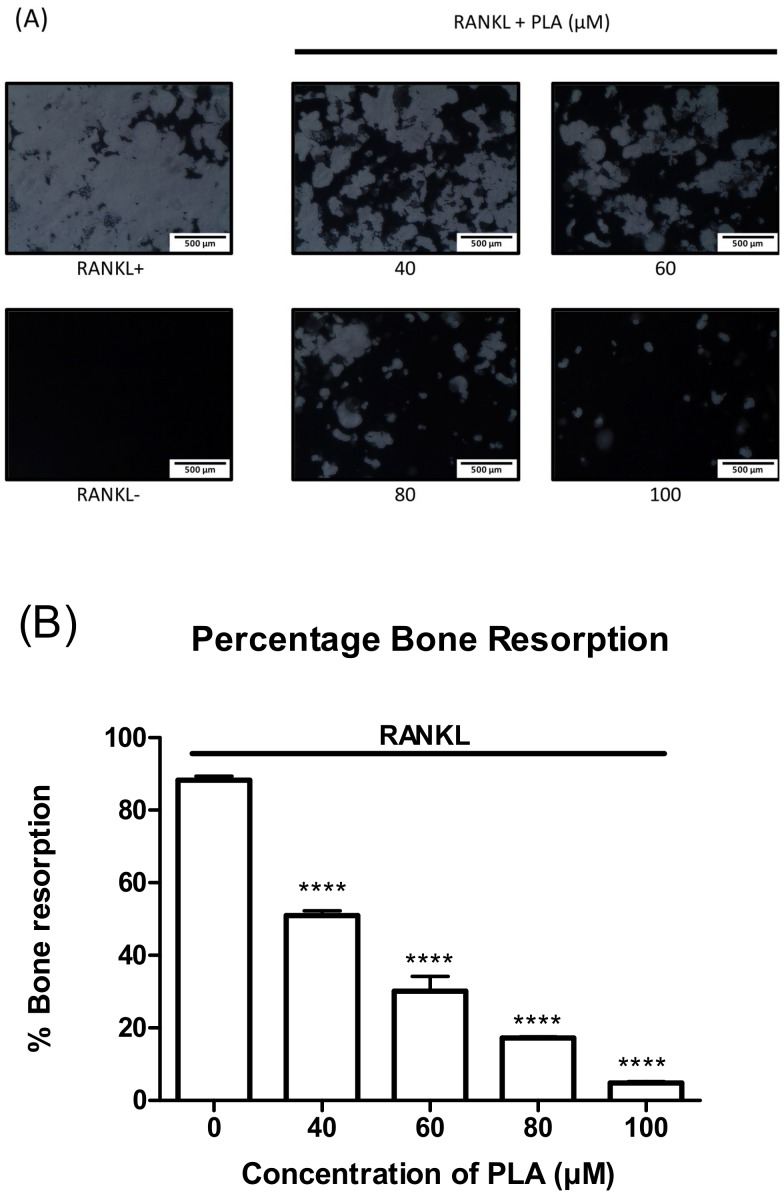
Effect of PLA on bone resorption. RAW264.7 macrophages were seeded onto bone mimetic plates in the presence of RANKL (15 ng/mL) and PLA (40–100 µM) for five days (**A**) Effect of PLA on bone resorption in RAW264.7 murine macrophages. The white areas are where the osteoclasts have resorbed the bone mimetic plate. (Scale bar = 500 µm); (**B**) Resorbed areas were quantified using ImageJ software and expressed as mean ± standard deviation. Results are shown as percentage of control. (**** *p* < 0.0001) compared to control.

**Figure 4:**
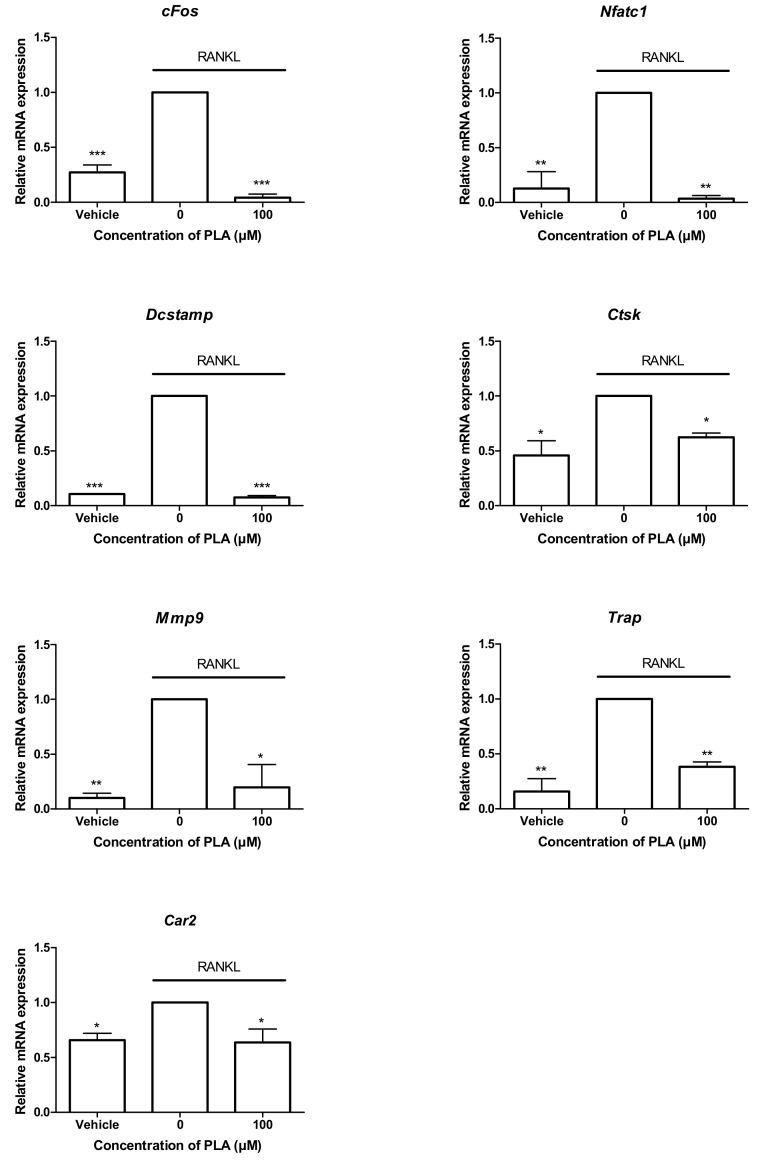
Effect of PLA on osteoclast specific gene expression. RAW264.7 murine macrophages were seeded at 1.5 × 10^4^ cells per well in the presence or absence of PLA (100 µM) in combination with RANKL (15 ng/mL) for five days. RNA was isolated and reverse transcribed into cDNA and relative expression of osteoclast specific genes was determined by quantitative-PCR. Results are expressed relative to the RANKL treated control. (* *p* < 0.05; ** *p* < 0.01; *** *p* < 0.001).

RAW264.7 murine macrophages were seeded onto a 24-well osteoassay plate coated with a layer of bone mimetic substrate. The cells were exposed to RANKL alone or in combination with PLA for 5 days. A decrease in bone resorption was observed with an increase in PLA concentration (Figure 3A). ImageJ software was used to quantify the percentage of bone resorption (Figure 4B). Decreases in bone resorption were shown to be statistically significant.

### 3.5. PLA Suppresses the Expression of Osteoclast-Specific Gene Expression

The binding of RANKL to the RANK receptor induces the expression of downstream signalling molecules that play a major role in the formation of osteoclasts as well as the bone resorbing function of mature osteoclasts. PLA (100 µM) treatment significantly reduced the expression of all osteoclast-specific genes tested in this study (Figure 4).

### 3.6. PLA Inhibits RANKL-Induced Activation of NF-κB and MAPK Pathways

**Figure 5:**
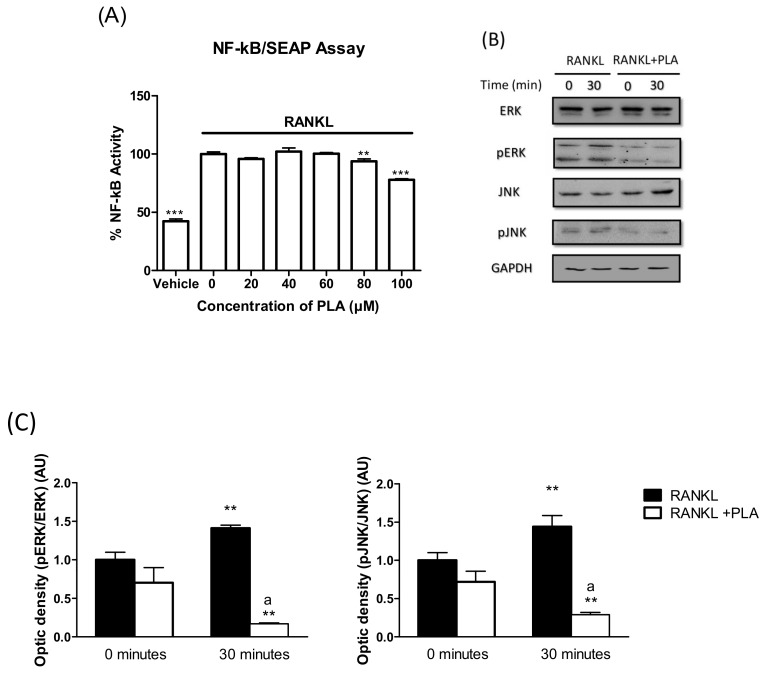
(**A**) RAW264.7 murine macrophages were transfected with nuclear factor kappa B (NF-κB)-SEAP reporter plasmid and exposed to RANKL (35 ng/mL) with or with PLA (20–100 µM) in serum-free selection media for 24 h. Secreted embryonic alkaline phosphatase (SEAP) reporter assay was performed as per the manufacturer’s instructions. Results are expressed as percent of positive control. (** *p* < 0.01; *** *p* < 0.001); (**B**). RAW264.7 cells were seeded at 1 × 10^6^ cells per well. Cells were exposed to RANKL (15 ng/mL) and PLA (100 µM) for 30 min. Thereafter cell lysates were prepared and western blot was performed to determine the activation of mitogen activated protein kinase (MAPK) proteins (ERK and JNK). Glyceraldehyde 3-phosphate dehydrogenase (GAPDH) was used as a loading control; (**C**) Densitometry analysis of bands was conducted using ImageJ software. Results are expressed as mean ± standard deviation; *n* = 3 per group (** *p* < 0.01 vs. 0 min RANKL) (^a^ *p* < 0.001 vs. 30 min RANKL).

Activation of both the NF-κB and MAPK pathways is integral in the formation and function of osteoclasts. To elucidate the effects of PLA on RANKL-induced NF-κB activation, RAW264.7 macrophages were stably transfected with NF-κB-SEAP reporter plasmid. Cells were treated with PLA (20–100 µM) in combination with RANKL (35 ng/mL) for 24 h. PLA at 80 and 100 µM significantly reduced RANKL-induced NF-κB activation after 24 h (Figure 5A).

Western blotting was conducted to test the effects of PLA on the activation of the MAPKs: JNK and ERK. After pre-exposing cells to PLA (100 µM) or vehicle for 4 h, cells were treated with RANKL (15 ng/mL) for 30 min. Vehicle control cells showed an increase in the phosphorylated form of the MAPK proteins, indicating activation of these proteins (Figure 5B). PLA suppressed the phosphorylation of both ERK and JNK, indicating inhibition of these pathways (Figure 5C).

### 3.7. Induction of Apoptosis by PLA in Mature Osteoclasts

**Figure 6:**
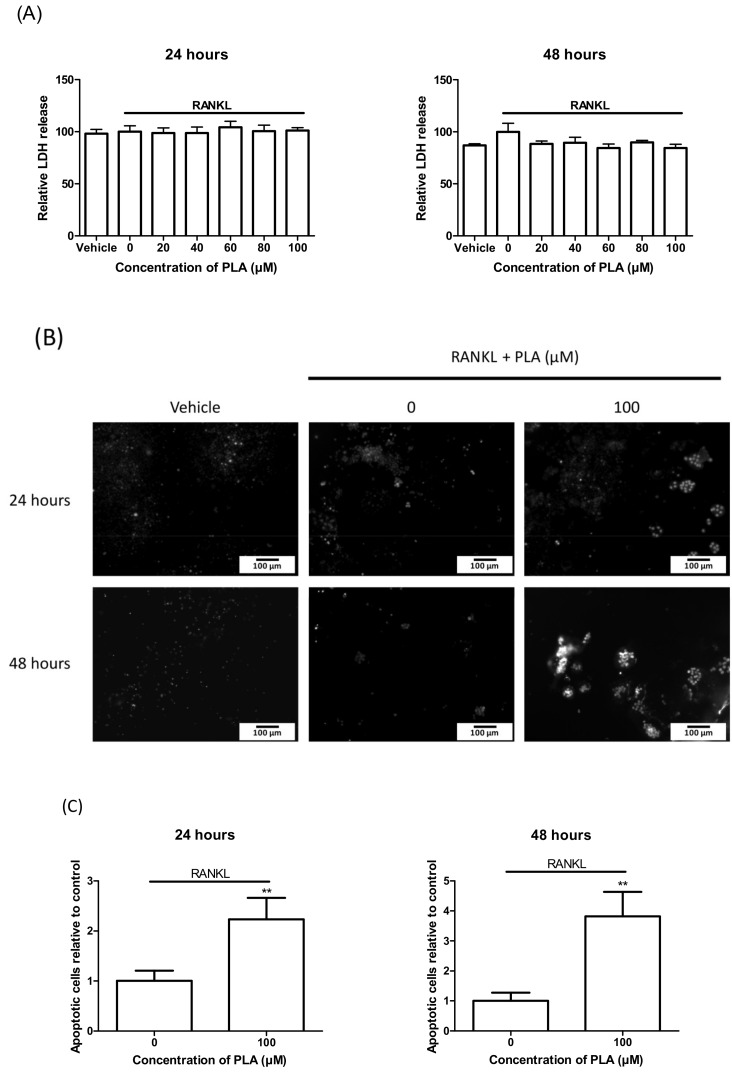
RAW 264.7 murine macrophages were matured in 96-well plates for 5 days followed by exposure to PLA (100 µM) for 24 h or 48 h. (**A**) Lactate dehydrogenase (LDH) results obtained 24 h and 48 h after treatment with PLA. Results are shown as percentage of positive control; (**B**) Hoechst staining performed on mature osteoclasts to visualize nuclear condensation and fragmentation. Scale bar = 100 µm; (**C**) Number of cells with nuclear fragmentation were counted. Results are expressed relative to the control. (** *p* < 0.01).

To elucidate the action of PLA on mature osteoclasts, RAW264.7 murine macrophages were differentiated in sterile 96-well plates. LDH release for necrosis and Hoechst staining for apoptosis was performed after 24 h and 48 h exposure to PLA. There was no significant increase in LDH release after 24 h or 48 h (Figure 6A). An increase in nuclear condensation and fragmentation was detected in the PLA exposed cells compared to the control (Figure 6B,C).

## 4. Discussion

Fatty acids (FAs) are key dietary nutrients crucial for health and well-being. FAs are commonly found in nuts, oils, seafood, and various other widely consumed food sources. Several studies have focused on the importance of unsaturated FAs in bone metabolism [8,10,11,28,29,30,31]. Chronic inflammatory diseases are known to increase the risk of bone fractures, as pro-inflammatory cytokines amplify the formation of osteoclasts [32]. Therefore, targeting osteoclast formation with anti-inflammatory compounds has been used as a strategy to combat bone diseases characterized by excessive osteoclast activity. For this reason, the effects of ω-3 long-chain polyunsaturated FAs (LCPUFAs) in particular have been studied in osteoclasts, as they are known to exert anti-inflammatory effects. Palmitoleic acid (PLA), an omega-7, 16-carbon monounsaturated FA (MUFA) is also known to possess anti-inflammatory effects [18]; however, the role of PLA on bone health remains unexplored. This study sought to investigate the effects of PLA on differentiating and mature osteoclasts in RAW264.7 murine macrophages to determine if PLA exerts any anti-osteoclastogenic potential.

In this study, the RAW264.7 murine macrophage model was used for osteoclastogenesis. RAW264.7 macrophages fuse and differentiate into mature resorbing osteoclasts in the presence of RANKL and are therefore suitable for studying in vitro effects on RANKL-induced osteoclast formation and activity [33]. Our findings show that PLA inhibited RANKL-induced osteoclastogenesis in RAW264.7 murine macrophages. Inhibition of osteoclast formation was accompanied by changes in morphology. During osteoclast differentiation, cytoskeletal rearrangement leads to the formation of actin rings. These actin rings play a crucial role in maintaining the cell structure, which is important for resorption to occur [34]. In this study, we found that actin ring formation was decreased by PLA. This was accompanied by a significant reduction in the osteolytic ability of osteoclasts derived from RAW264.7 macrophages. The decrease in actin ring formation and resorption was most likely due to inhibition of osteoclast formation by PLA. However, PLA was shown to downregulate genes that play a role in the fusion of osteoclast precursors (*Dcstamp*, dendritic cell-specific transmembrane protein) and in the resorptive process (*Ctsk, Mmp9, Trap, Car2*). This was most likely due to the reduction in *cFos* and *Nfatc1*, which are crucial for the expression of osteoclast specific characteristics [35]. NFATc1 is known as the master regulator of osteoclast formation and function and its activation is a key point in the RANKL signalling cascade.

RANKL-induced activation of *cFos* and *Nfatc1* is downstream of the activation of the NF-κB and MAPK pathways [36]. NF-κB deficient mice have been shown to have no osteoclasts and develop severe osteopetrosis [37]. The MAPKs, p-38 [38], JNK1 [39], and ERK have all been shown to be activated when RANK is stimulated and are critical to osteoclast formation [36]. Therefore, targeting NF-κB and MAPK may lead to decreases in osteoclastogenesis and bone loss. Rahman et al. have shown that the ω-3 LCPUFAs, docosahexaenoic acid (DHA), and eicosapentaenoic acid (EPA) can inhibit osteoclast formation through inhibition of NF-κB nuclear translocation and p-38 expression in RAW264.7 murine macrophages [12]. More recent studies on DHA have shown inhibition of JNK, ERK, and p-38 activation as well as NF-κB activity in mouse bone marrow macrophages [11]. Similar to results on ω-3 LCPUFAs, we found that PLA inhibited NF-κB activity as well as the activation of the MAPKs, JNK and ERK. Supplementation with high doses of EPA and DHA has been shown to decrease bone loss associated with breast cancer [40]. Furthermore, diets rich in ω-3 LCPUFA have been associated with increased bone mineral density and peak bone mass [30]. Our results suggest that PLA may possess bone protective effects similar to ω-3 LCPUFAs.

Additionally, we found that, not only did PLA affect osteoclast formation, it further induced apoptosis in mature osteoclasts. Nuclear condensation and fragmentation are key markers of apoptosis [41]. We report that PLA induced both nuclear condensation and fragmentation in matured osteoclasts indicating apoptosis. Quantification of cells with nuclear fragmentation revealed that exposure to PLA significantly increased the number of apoptotic cells. This result is again similar to results reported on bone marrow macrophages exposed to DHA [11]. When cells are necrotic, their plasma membranes become permeabilized and allow cellular components to leak out [27]. Lactate dehydrogenase (LDH), a soluble cytoplasmic enzyme, is one such component known to leak out of these damaged cells [26]. We found that PLA did not significantly increase LDH release, indicating that the cells were not necrotic. These results indicate that PLA induced programmed cell death in mature osteoclasts.

## 5. Conclusions

This study reveals the potential anti-osteoclastogenic properties of PLA for the first time. Our findings show that treatment with PLA inhibits RANKL-induced formation of osteoclasts and interfered with the expression of osteoclast-specific genes in vitro. PLA inhibited the activation of the NF-κB and MAPK pathways, offering a possible mechanism of action for its anti-osteoclastogenic effects. PLA further stimulated apoptosis in mature osteoclasts. Therefore, this study provides evidence that PLA may be developed as a nutraceutical for the treatment of diseases characterized by excessive osteoclast formation.

## Figures and Tables

**Table 1:** Primers that were used in this study.

Gene	Forward Primer Sequence (5′–3′)	Reverse Primer Sequence (5′–3′)
***Gapdh***	GATGACATCAAGAAGGTGGTGAAGC	ATACCAGGAAATGAGCTTGACAAAG
***Mmp9***	GTCATCCAGTTTGGTGTCGCG	AGGGGAAGACGCACAGCTC
***Ctsk***	CTGGAGGGCCAACTCAAGA	CCTCTGCATTTAGCTGCCTT
***Trap***	CAGCTGTCCTGGCTCAA	GTAGGCAGTGACCCCGT
***cFos***	CCCATCGCAGACCAGAGC	ATCTTGCAGGCAGGTCGGT
***Dcstamp***	ATGACTTGCAACCTAAGGGCAAAG	GTCTGGTTCCAAGAAACAAGGTCAT
***Nfatc1***	GTGGAGAAGCAGAGCAC	ACGCTGGTACTGGCTTC
***Car2***	GAGTTTGATGACTCTCAGGACAA	CATATTTGGTGTTCCAGTGAACCA

*Gapdh*: glyceraldehyde 3-phosphate dehydrogenase; *Mmp9*: matrix metalloproteinase 9; *Ctsk*: cathepsin K; *Trap*: tartrate resistant acid phosphatase; *cFos*: Fos proto-oncogene; *Dcstamp*: dendritic cell-specific transmembrane protein; *Nfatc1*: cytoplasmic 1; *Car2*: carbonic anhydrase 2.
